# When time does not matter: cultures differ in their use of temporal cues to infer agency over action effects

**DOI:** 10.1007/s00426-023-01911-y

**Published:** 2024-01-11

**Authors:** Victoria K. E. Bart, Erdenechimeg Sharavdorj, Enerel Boldbaatar, Khishignyam Bazarvaani, Martina Rieger

**Affiliations:** 1 Department for Psychology and Sports Medicine, Institute of Psychology, UMIT Tirol - Private University for Health Sciences and Health Technology, Hall in Tirol, Austria; 2 National University of Mongolia, Ulaanbaatar, Mongolia; 3 Mongolian National University of Medical Sciences, Ulaanbaatar, Mongolia

## Abstract

Sense of agency (SoA) is the sense of having control over one’s own actions and through them events in the outside world. Sometimes temporal cues, that is temporal contiguity between action and effect, or temporal expectation regarding the occurrence of the effect are used to infer whether one has agency over an effect. This has mainly been investigated in Western cultures. However, Western and Eastern cultures differ in their time concepts and thus their usage of temporal cues may also differ. We investigated whether Western and Eastern cultures (Austrian vs. Mongolian students) use temporal cues differently. Participants performed adaption blocks in which actions were followed by immediate (immediate effect group) or by delayed (delayed effect group) effects. In subsequent test blocks the action–effect delay was varied and participants’ SoA over the effect was assessed. In Austrian students, the immediate effect group experienced more SoA for short action–effect delays, whereas the reverse was true for the delayed effect group. Thus, temporal expectation rather than temporal contiguity is used as predominant agency cue. In Mongolian students, SoA did not significantly differ between different action–effect delays in both groups, indicating that Mongolian students hardly rely on temporal cues. In conclusion, due to linear time concepts in Western cultures, the timing of an effect may be an important agency cue in Austrian students. However, due to cyclical time concepts in some Eastern cultures, it may be a less important agency cue in Mongolian students. Thus, the use of temporal agency cues is culture-dependent.

## Introduction

Sense of agency (SoA) refers to the sense of having control over one’s own actions and through them events in the outside world (Haggard & Chambon, 2012; Haggard & Tsakiris, 2009). SoA underpins many important characteristics of human societies (Haggard, 2017). For instance, it is strongly associated with beliefs in free will (Aarts & van den Bos, 2011) and enables attribution of social or legal responsibility, which is vital for a functioning society (Haggard & Tsakiris, 2009; Moore, 2016). Moreover, it is associated with the development of certain social-cognitive competences such as theory of mind (Zalla et al., 2015) or perspective taking (David et al., 2008). Further, disruptions of SoA, for instance caused by mental illnesses (e.g., schizophrenia, Jeannerod, 2009, or borderline personality disorder, Colle et al., 2020) heavily impact one’s quality of life. Thus, SoA seems to be a core feature of human existence. Correspondingly, it has been proposed that SoA is a universal concept that exists across different cultures (cf. Aarts et al., 2010; Bart et al., 2019). However, cultures differ in their underlying models of SoA (Markus & Kitayama, 2003) and the few studies which so far have assessed SoA across different cultures have observed not only similarities, but also differences, particularly with regard to agency cues that people rely on when inferring agency over effects (Aarts et al., 2010; Barlas & Obhi, 2014; Bart et al., 2019). It has been speculated that temporal cues, i.e., the time after which an action is followed by the effect, may be less likely used as agency cues in Eastern cultures (Mongolian students) compared to Western cultures (Austrian students) due to cultural differences in time concepts (Bart et al., 2019). However, so far this has not been systematically investigated. Thus, the aim of the present study was to investigate whether Austrian and Mongolian students differ in their use of temporal cues to infer agency over effects.

Per definition, sense of agency is a sense of having control and thus not necessarily a flawless reproduction of the objective reality but rather a subjective experience (cf. Moore, 2016). According to cue integration models people infer SoA by weighing various possible explanations for actions and their effects (Farrer et al., 2013; Synofzik et al., 2008; Synofzik et al., 2009; Synofzik et al., 2013; for a review see Moore & Fletcher, 2012). For instance, different sensorimotor cues (e.g., a comparison between internally predicted and actual sensory consequences of an action, Krugwasser et al., 2019; Sato & Yasuda, 2005; Spengler et al., 2009; Synofzik et al., 2013), cognitive cues (e.g., one’s own intentions, beliefs, expectations, or contextual information, Farrer & Hupe, 2013; Lafleur et al., 2020; Moore et al., 2009a, 2009b; Pronin et al., 2006; Wegner & Wheatley, 1999), and affective cues (e.g., affective valance of the effect of an action, emotional state of the actor, Gentsch & Synofzik, 2014) may be weighted and integrated according to their reliability in a given situation. The cues contribute to one’s judgement whether oneself or someone else is more likely responsible for a certain action or effect (Moore & Fletcher, 2012; Synofzik et al., 2009).

However, so far, the majority of those cues and their contribution to SoA have been investigated in Western cultures. The few studies, which have investigated cross-cultural differences in SoA, propose that some of those cues are used in a similar way in Eastern cultures (i.e., are used universally), whereas the use of others may vary depending on culture (Aarts et al., 2010; Barlas & Obhi, 2014; Bart et al., 2019). One cue that seems to be used universally is action–effect congruency. If internally predicted sensory consequences of an action (based on forward models, Wolpert, 1997; Wolpert & Flanagan, 2001) and actual sensory consequences of the action match, SoA is enhanced in both Western and Eastern cultures (Bart et al., 2019). A further universal agency cue is the affective valence of an effect. It has been observed that SoA is higher for positive than for negative effects in both, Western and Eastern cultures (at least for explicit SoA ratings, Barlas & Obhi, 2014; Bart et al., 2019). Differences in the use of agency cues have been observed regarding temporal cues, presumably due to differences in time concepts between cultures. Western cultures (Austrian students) rely more strongly on temporal cues, i.e., the time after which an action is followed by the effect, than Eastern cultures (Mongolian students) when inferring SoA (Bart et al., 2019).

One temporal cue that people from Western cultures rely on, is temporal contiguity, that is sense of agency is high if an effect appears immediately after the action. This may be explained by perceived causality, which is closely linked to SoA (De Vignemont et al., 2004; Moore et al., 2009a, 2009b). The shorter the delay between one’s own action and the resulting effect, the more likely the action is perceived as the cause of the effect (Greville & Buehner, 2010; Reed, 1992; Shanks et al., 1989), thereby increasing one’s SoA (Farrer et al., 2013; Metcalfe et al., 2010; Ruess et al., 2017). Similar results have been obtained in Eastern cultures by investigating Japanese participants (Kawabe, 2013; Sato & Yasuda, 2005; Wen et al., 2015), but in these studies results were not directly compared to Western cultures.

Even though most of the time actions are immediately followed by effects, people sometimes experience situations in which effects are delayed (e.g., waiting for the energy saving light bulb to brighten up, cf. Buehner & May, 2004). Nevertheless, they may still experience causality (Buehner & May, 2002, 2003, 2004; Buehner & McGregor, 2006) and SoA (Haering & Kiesel, 2015, 2016). Thus, temporal contiguity may not be the only temporal cue driving SoA. Instead, temporal expectation may be another agency cue. That is, sense of agency is high for effects, whose timing matches prior experiences with that effect. To the best of our knowledge, temporal expectation has so far only been investigated in Western cultures.

According to the ideomotor theory, the repeated execution of actions results in a bidirectional association of these actions with their effects (Hommel et al., 2001; Prinz, 1987). Therefore, planning or performing these actions entails automatic expectations/predictions of their effects and intended effects activate the corresponding actions (Elsner & Hommel, 2001; Rieger, 2007; for a review see Kunde et al., 2007). It has been observed that the temporal relation between actions and effects, that is the delay between an action and its effect, becomes integrated in action–effect associations and is automatically retrieved during planning and performing actions (Dignath & Janczyk, 2017; Dignath et al., 2014). Thus, based on previous experiences about usual action–effect delays (Haering & Kiesel, 2012; Walsh & Haggard, 2013), predictions about the timing of certain effects in relation to the actions that produces them are formed. Such predictions weaken (Buehner & May, 2002) or even override (Buehner & May, 2004) the influence of temporal contiguity on perceived causality, and may thereby also affect SoA. Correspondingly, it has been observed that SoA is higher when previous experiences regarding the appearance of effects match the actual timing of effects (Haering & Kiesel, 2015, 2016). Thus, in some instances temporal expectation may be a more reliable agency cue than temporal contiguity (Haering & Kiesel, 2015; but see Ruess et al., 2017).

From the perspective of time perception, there are two different mechanisms that may explain why people experience SoA in cases in which temporal contiguity is violated (see also Haering & Kiesel, 2015 for an extensive discussion of this topic). First, it has been observed that in instances in which one experiences SoA over an effect, the effect is perceived as slightly earlier in time (known as the temporal binding effect, cf. Haggard et al., 2002; Tsakiris & Haggard, 2003; see Moore & Obhi, 2012 for a review). Accordingly, participants who experience SoA over a delayed effect (because the actual delay matches their expected delay) may no longer perceive the delay as a violation of temporal contiguity, because the effect is perceived as earlier/the action–effect delay is perceived as shorter than it is due to temporal binding (Haering & Kiesel, 2015). Second, in a similar way as temporal binding, temporal recalibration (cf. Stetson et al., 2006) may play a role (Haering & Kiesel, 2015). An effect that appears at a consistent delay after an action, is perceived as a consequence of this action and thus, action and effect are bound into a single event. Once it is interpreted as a consequence of one’s action, the brain recalibrates timing judgments such that the perceived timing of an effect shifts toward the action to fit prior assumptions that effects normally follow actions without delay (Stetson et al., 2006). Thus, again the delay between action and effect is perceived as shorter and therefore may no longer be perceived as violation of temporal contiguity (Haering & Kiesel, 2015).

Considering the importance of time and time perception for the perception of causality and SoA (e.g., Haering & Kiesel, 2015, 2016), it seems reasonable that differences in time concepts might alter perceived causality and correspondingly SoA (Widlock, 2014; Vuorre, 2017). Large discrepancies in time conception and time perception have been observed between Western cultures, e.g., in American or European countries and Eastern cultures, e.g., in Asian countries (Block et al., 1996; Briley, 2009; Brislin & Kim, 2003; Ezzell, 2002; Fulmer et al., 2014; Leung et al., 2011; Levine, 1997/2006). For instance, in Western cultures time-efficiency and punctuality are important, people are anxious not to waste their time, persons who are frequently late leave an overall negative impression, and life follows a rather fast pace (Brislin & Kim, 2003; Levine et al., 1980). In contrast, in some Eastern cultures punctuality is rated as less important, definitions of “late” or “early” are more flexible, and life follows a rather slow pace (Brislin & Kim, 2003; Levine et al., 1980). Further, whereas Western cultures have usually linear time concepts, in which time moves in one direction from the past to the present to the future, some Eastern cultures have cyclical or reversible time concepts, with emphasis on repetition or reoccurrence of events (e.g., seasonal cycle, cycle of day and night) (Brodowsky et al., 2008; Dahl, 1995; Levine, 1997/2006; Widlok, 2014).

Thus, one may speculate that temporal cues to infer SoA are used differently across different cultures (cf. Bart et al., 2019 for such a discussion). In particular, in linear time concepts, in which one cannot go back in time, once an event has passed, it is irretrievable. Therefore, people may pay attention to the timing of events. In contrast, in cyclical time concepts, in which events repeat themselves, an event that has passed will come back in the future. Therefore, people may not pay much attention to the timing of events. Accordingly, the timing of events (in the present context the delay at which an effect occurs after the action) may also play a less important role when inferring causality (Widlock, 2014) and thus may be a less reliable agency cue in Eastern cultures*.* However, so far, this has hardly been investigated. Even though some studies investigating Eastern samples (Japanese participants) could show that temporal contiguity is used as agency cue (Kawabe, 2013; Sato & Yasuda, 2005; Wen et al., 2015), they did not investigate whether this cue is relied on more or less strongly compared to Western cultures. In a recent study of Bart et al. (2019), the use of temporal cues was directly compared between Western (Austrian students) and Eastern (Mongolian students) cultures. In that study, decreasing SoA was observed with increasing action–effect delay only in Austrian students, whereas SoA was similarly pronounced with all action–effect delays in Mongolian students, indicating that the temporal occurrence of the effect is not used as agency cue in Mongolian students. However, in that study different temporal cues (temporal contiguity vs. temporal expectation) could not be dissociated as at the shortest delay (100 ms) temporal contiguity was high and expected timing (in a prior learning phase effects were presented without delay, so participants may have expected that the effect occurs immediately) corresponded most to the actual timing.

Thus, the aim of the present study was to investigate whether Western cultures (Austrian students) and Eastern cultures (Mongolian students) differ in their use of temporal cues (i.e., temporal contiguity, temporal expectation) to infer SoA. To this end, we adapted an experimental paradigm introduced by Haering and Kiesel (2015) that allows to differentiate between the contribution of temporal contiguity and temporal expectation to SoA. Austrian and Mongolian students first performed adaption blocks in which their action was followed either by immediate effects (immediate effect group) or by delayed effects (delayed effect group). Thus, depending on group different temporal expectations regarding the appearance of the effect were induced. Afterwards, participants performed test blocks, in which the delay between action and effect was varied and participants were asked to rate how much control they experienced over the effect.

For Austrian students, we expected to replicate the results of previous studies according to which a match between the actual and expected timing of an effect overrides the influence of temporal contiguity on SoA (cf. Haering & Kiesel, 2015, 2016). Thus, we expected that control ratings increase the more the timing of an effect matches its usual timing. Accordingly, for the immediate effect group control ratings should be highest if effects occur immediately, but for the delayed effect group control ratings should be highest if effects occur with the same delay as in the adaption blocks. If temporal cues are not or to a lesser extent used to infer SoA over effects in Mongolian students, control ratings in Mongolian students should be less affected by the delay between action and effect in both the immediate effect group and the delayed effect group.

## Methods

### Participants

A total of 293 participants took part in the study. 140 participants were students of the UMIT TIROL—Private University for Health Sciences and Health Technology in Austria (nationality: 51 from Germany, 8 from South Tyrol in Italy, 81 from Austria; sex: 111 female, 29 male; handedness: 120 right, 19 left, 1 ambidextrous; age in years: *M* = 22.6, *SD* = 3.11). They were randomly assigned to one of two groups, which differed in the temporal expectation that was induced regarding the appearance of the effect (immediate effect group: *N* = 70, delayed effect group: *N* = 70). 153 participants were students of the National University of Mongolia (nationality: all from Mongolia; sex: 128 female, 25 male; handedness: 138 right, 9 left, 6 ambidextrous; age in years: *M* = 19.8, *SD* = 1.86), which were also randomly assigned to the two different groups (immediate effect group: *N* = 74, delayed effect group: *N* = 79).

We used GLIMMPSE (https://glimmpse.samplesizeshop.org; Kreidler et al., 2013) to calculate the required sample size for the three-way interaction of interest (culture × temporal expectation × delay). Statistical significance was set at *p* < 0.05. Means and within participant variability were estimated based on previous studies (Bart et al., 2019; Haering & Kiesel, 2015). The required sample size to achieve a power of 0.85 is a minimum of 228 participants, that is a minimum of 57 participants for each between-participants condition.

All procedures in the present study were in accordance with the 1964 Helsinki declaration and its later amendments. The study was approved by the local ethics committee and participants gave informed consent. Participants performed the experiment for course credit.

### Material and procedure

Participants performed the experiment in groups of maximal 30 participants in the computer labs of the respective universities. Participants were seated at desks approximately 50 cm in front of a computer screen. In Austria HP z23i monitors (screen: 23″, vertical refresh rate: 60 Hz, resolution: 1920 × 1080 pixels) and in Mongolia Intel i3 monitors (screen: 19″, vertical refresh rate: 60 Hz, resolution: 1366 × 768 pixels) were used. The experiment was programmed with the open-source experimental software OpenSesame (https://osdoc.cogsci.nl; Mathôt et al., 2012).

Participants completed adaption blocks and test blocks. In adaption blocks, each trial started with the presentation of a grey cloud (RGB: 127, 127, 127; size: 7.4 cm × 4.4 cm) on a black background in the center of the screen. Participants were instructed to press the spacebar at the keyboard with the index finger of their left hand as soon as the cloud appeared. This response was followed by the presentation of a yellow flash (RGB: 255, 255, 73; size: 5.3 cm × 10.95 cm) for 100 ms, which was either presented immediately (delay of 0 ms, immediate effect group) or after a delay of 600 ms (delayed effect group). After an inter-trial-interval of 2000 ms the next trial started. At the beginning of each adaption block participants were informed that during the following block their response causes the flash. Each adaption block consisted of 50 trials.

In test blocks, the trial procedure was similar to the adaption blocks except for the following differences: Whereas a stable action–effect delay was used in adaption blocks, the flash was presented after a variable delay (0 ms, 150 ms, 300 ms, 450 ms, or 600 ms) in test blocks. As in the adaption blocks, participants' response always caused the flash. To create uncertainty about the extent of one’s control, participants were informed at the beginning of each test block that during the following block their response usually causes the flash, but that in some trials the flash is automatically generated by the computer independent of their response. In every trial 500 ms after the disappearance of the flash participants were asked to indicate via mouse click on a visual analog scale (14 cm) from “not at all” (0%) to “complete control” (100%) how much control they experienced over the flash. After an inter-trial-interval of 2000 ms the next trial started. Each test block consisted of 50 trials. The flash occurred in 30 trials with its usual delay (either 0 ms or 600 ms depending on the group) and with any of the other delays in 5 trials per test block. The original instructions (in German and Mongolian), their English translation and the used stimuli can be accessed from https://osf.io/kuemv/.

Participants performed three initial adaption blocks. Afterwards they performed five test blocks, each separated by an adaption block.^1^ The whole experiment lasted approximately one hour.

### Data analysis

The data are available at the open science framework, https://osf.io/kuemv/. We calculated mean control ratings per participant separately for each delay. As mentioned above, in each test block the flash occurred in 30 trials with its usual delay (either 0 ms or 600 ms depending on the group) and with any of the other delays in 5 trials per test block. To ensure that the mean control ratings for the different delays are based on an equal number of trials, we randomly chose five trials out of the 30 trials with the usual delay per test block to calculate the mean control ratings.^2^ This resulted in overall 25 trials per delay (five test blocks with each five trials per delay).

To investigate whether control ratings differed between cultures depending on temporal expectation and delay, an ANOVA with the between-participants factors culture (Austria, Mongolia) and temporal expectation (immediate effect group, delayed effect group) and the within-participants factor delay (0 ms, 150 ms, 300 ms, 450 ms, 600 ms) was performed on the control ratings. If Mauchly’s test indicated that the assumption of sphericity was violated, Greenhouse–Geisser corrected *F*-values, *p*-values, and Greenhouse–Geisser’ε are reported. Post hoc comparisons were conducted using paired *t*-tests. Significance values were adjusted for multiple testing using Sidak correction. When several post hoc comparisons are reported together, minimum (*p*_min_) or maximum *p*-values (*p*_max_) are reported.

Further, to assess the correlation coefficients between delay and control rating and the corresponding regression slopes, we computed regression analyses predicting the control ratings for the delays between 0 and 600 ms, separately for each participant. The resulting regressions slopes and correlation coefficients of each participant’s regression function were than averaged over participants, separately for the different between-participants conditions. To average correlation coefficients, Fishers’ z transformation and back-transformation were used.

## Results

### Differences in control ratings depending on culture, temporal expectation, and delay

**Fig. 1:**
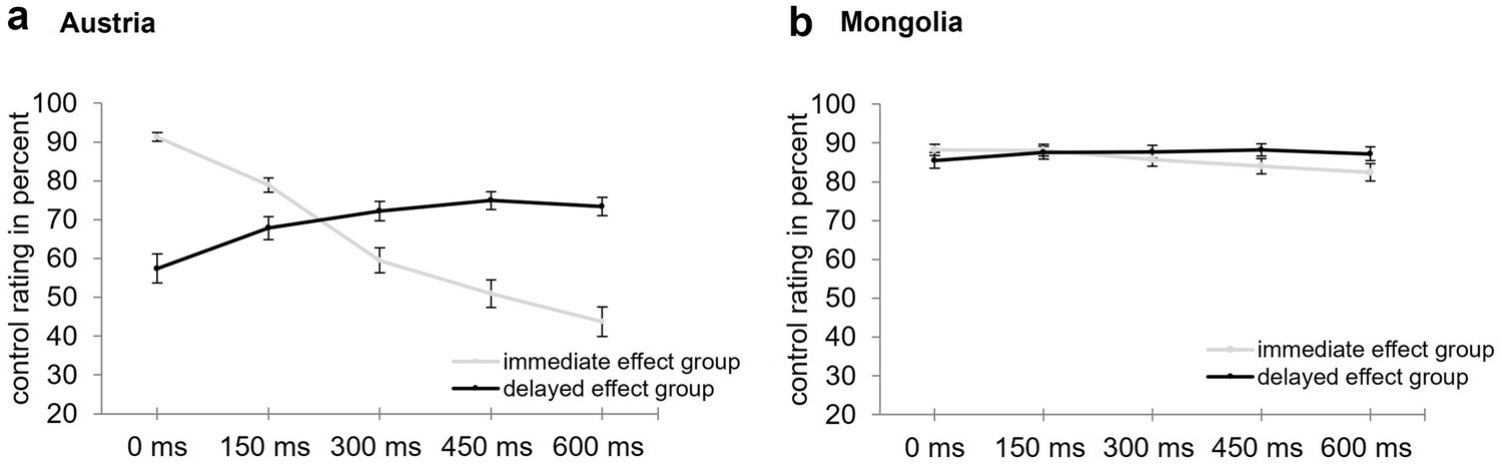
Means and standard errors of control ratings depending on temporal expectation (immediate effect group, delayed effect group) and delay (0 ms, 150 ms, 300 ms, 450 ms, 600 ms), separately for the Austrian (**A**) and Mongolian (**B**) participants

Means and standard errors of control ratings, separately for Austrian and Mongolian students can be seen in Fig. 1. A significant main effect of culture, *F*(1, 289) = 94.7, *p* < 0.001, η^2^_p_ = 0.25, indicated higher control ratings in Mongolian students (*M* = 86.5, *SE* = 1.38) than in Austrian students (*M* = 67, *SD* = 1.44). The significant main effect of delay, *F*(1.4, 406.8) = 33.9, *p* < 0.001, η^2^_p_ = 0.11, the significant interaction between culture and delay, *F*(1.4, 406.8) = 19, *p* < 0.001, η^2^_p_ = 0.06, and the significant interaction between temporal expectation and delay,* F*(1.4, 406.8) = 123, *p* < 0.001, η^2^_p_ = 0.3, were modified by a significant interaction between culture, temporal expectation, and delay, *F*(1.4, 406.8) = 76.5, *p* < 0.001, η^2^_p_ = 0.21. In Austrian students in the immediate effect group, control ratings were highest at the 0 ms delay and significantly decreased with increasing delay (all *p* < 0.001). In contrast, in the delayed effect group control ratings were higher at the 600 ms, 450 ms, 300 ms, and 150 ms delay than at the 0 ms delay (all *p* < 0.001) and higher at the 450 ms and 300 ms delay than at the 150 ms delay (*p*_max_ = 0.006). All other comparisons were not significant (*p*_min_ = 0.067). In Mongolian students no significant differences between delays were observed neither in the immediate effect group (*p*_min_ = 0.091) nor in the delayed effect group (*p*_min_ = 0.73). The main effect of temporal expectation, *F*(1, 289) = 2.1, *p* = 0.15, η^2^_p_ = 0.01, and the interaction between culture and temporal expectation, *F*(1, 289) = 0.49, *p* = 0.49, η^2^_p_ = 0.002, were not significant.

### Correlation coefficients and regression slopes between control ratings and delay depending on culture and temporal expectation

In Austrian students, a negative correlation coefficient between delay and control rating,* r* =  − 0.95, *p* < 0.001 with a regression slope of − 0.08 was observed in the immediate effect group, whereas a positive correlation coefficient, *r* = 0.32, *p* = 0.007 with a regression slope of 0.03 was observed in the delayed effect group. In Mongolian students, again the correlation coefficient between delay and control rating was negative, *r* =  − 0.34, *p* = 0.003 with a regression slope of − 0.01 in the immediate effect group, whereas it was positive, but not significant, *r* = 0.16, *p* = 0.17 with a regression slope of 0.003 in the delayed effect group.

To check whether the size of the correlation coefficients and regression slopes differed between cultures and depending on temporal expectation, we inverted the correlation coefficients and regression slopes in the delayed effect group by multiplying them with − 1 (cf. Haering & Kiesel, 2015) and computed ANOVAs with the between-participants factors culture (Austria, Mongolia) and temporal expectation (immediate effect group, delayed effect group). The results of those ANOVAs on the correlation coefficients and regression slopes can be seen in Table 1. The significant main effect of culture indicated that correlation coefficients and regression slopes were higher in Austrian students than in Mongolian students. The significant main effect of temporal expectation was modified by a significant interaction between culture and temporal expectation. Regression slopes and correlation coefficients were higher in the immediate effect group than in the delayed effect group in Austrian students (*p*_max_ < 0.001), but no significant differences were observed in Mongolian students (*p*_min_ = 0.24).

**Table 1:** Results of the ANOVAs with the between-participants factors culture (Austria, Mongolia) and temporal expectation (immediate effect group, delayed effect group) on correlation coefficients and regression slopes

	df_1_, df_2_	*F*	*p*	η^2^_p_
*Correlation coefficients*				
Culture	1, 289	47.2	< 0.001	0.14
Temporal expectation	1, 289	50.6	< 0.001	0.15
Culture × temporal expectation	1, 289	29.8	< 0.001	0.09
*Regression slopes*				
Culture	1, 289	89	< 0.001	0.24
Temporal expectation	1, 289	39.8	< 0.001	0.12
Culture × temporal expectation	1, 289	22.9	< 0.001	0.07

The ANOVA on the correlation coefficients was conducted on Fisher’s z-transformed correlation coefficients

## Discussion

We aimed to investigate whether Western cultures (Austrian students) and Eastern cultures (Mongolian students) differ in their use of temporal agency cues, that is temporal contiguity between action and effect and temporal expectation regarding the occurrence of the effect. Participants performed adaption blocks in which actions were followed by immediate (immediate effect group) or by delayed (delayed effect group) effects. In subsequent test blocks the action–effect delay was varied and participants were asked to rate how much control they experienced over the effect. In Austrian students control ratings were higher for short action–effect delays than for long action–effect delays in the immediate effect group, whereas the reverse was the case in the delayed effect group. Further, correlation coefficients between delay and control rating and corresponding regression slopes were negative for the immediate effect group and positive for the delayed effect group. Correlation coefficients and regression slopes were higher in the immediate effect group than in the delayed effect group. In Mongolian students, control ratings did not significantly differ between different action–effect delays neither in the immediate effect group nor in the delayed effect group. Further, even though correlation coefficients and regression slopes were in the same direction as in Austrian students, they did not significantly differ between the immediate and delayed effect group and only the correlation coefficient in the immediate effect group was significantly different from zero.

Significant higher control ratings for short than for long action–effect delays in the immediate effect group and the reverse data pattern in the delayed effect group in Austrian students indicate that SoA declines the more the actual timing of an effect deviates from its usual timing. This is supported by correlation coefficients between delay and control rating and the corresponding regression slopes, which were negative for the immediate effect group and positive for the delayed effect group. Those results replicate previous studies (Haering & Kiesel, 2015, 2016) and indicate that temporal expectation regarding the occurrence of the effect is used as agency cue in Austrian students. The use of temporal expectation as an agency cue may be explained by the ideomotor theory. It has been observed that in addition to the formation of bidirectional associations between actions and effects (Hommel et al., 2001; Prinz, 1987), the delay between them is integrated in the action–effect associations (Dignath & Janczyk, 2017; Dignath et al., 2014). This action–effect delay is retrieved when performing an action (Dignath & Janczyk, 2017; Dignath et al., 2014) and SoA is enhanced in case of a match with the actual delay (Haering & Kiesel, 2015, 2016). Further, as already outlined in the introduction, due to different time perception processes such as temporal binding (Haggard et al., 2002) or temporal recalibration (Stetson et al., 2006) the delay between action and effect may have been perceived as shorter than it actually was, such that participants did perceive delayed effects not so much as a violation of temporal contiguity in the delayed effect group (cf. Haring & Kiesel, 2015).

We further observed that correlation coefficients and regression slopes were smaller in the delayed effect group than in the immediate effect group in Austrian students (see also Fig. 1 for the clearly flatter curve in the delayed effect group than in the immediate effect group). Thus, deviations from the expected timing predicted control ratings less well in the delayed effect group than in the immediate effect group, suggesting that SoA ratings were not only determined by temporal expectation but presumably also by temporal contiguity. Those two cues may have counteracted each other in the delayed effect group resulting in an overall flatter curve.

This stands in contrast to the results of Haering and Kiesel (2015), who did not observe differences in correlation coefficients and regression slopes between groups and thus concluded that SoA is not driven by temporally contiguity, but solely by temporal expectation. So far, we can only speculate why our results differ. Hearing and Kiesel (2015) used auditory effects and delays ranging from 0 to 250 ms, increasing in increments of 50 ms. In the present study visual effects and delays ranging from 0 to 600 ms, increasing in increments of 150 ms, were used.^3^ It may be possible that in instances in which action and effect are already very close together in time (temporal contiguity is high, e.g., at rather short delays of up to 250 ms), a match between the expected and actual timing of an effect is the only agency cue used to infer SoA. In contrast, in instances in which action and effect have a larger temporal distance (e.g., delays longer than 250 ms), participants may attempt to take both cues, temporal contiguity (because in daily life people often experience that effects follow their actions immediately) and temporal expectation (because of previous experience with a particular effect) into account. Those cues may counteract each other. This would also be in line with studies, which investigated the influence of temporal contiguity and temporal expectation on causality and observed that depending on for instance contextual information (e.g., how reasonable is it for the effect to occur late), one or the other cue may be relied on more strongly (Buehner & May, 2002, 2004). Nevertheless, even though both cues seem to affect SoA in Austrian students, the results of the present study are in favor of the assumption that temporal expectation is used as the more predominant cue.

Interestingly, we observed a clearly distinct data pattern in Mongolian students. Control ratings did not differ significantly depending on delay, neither in the immediate effect group and nor in the delayed effect group. Further, even though correlation coefficients between control rating and delay and regression slopes were in the same direction as in Austrian students, they were lower in Mongolian than in Austrian students, did not significantly differ between the immediate and delayed effect group and the correlation coefficient was not significant in the delayed effect group. One may wonder whether Mongolian students, in contrast to Austrian students, did not believe the instructions that in some instances the computer automatically generates the flash and thus overall had a very high sense of agency in all conditions. However, in an earlier study with a similar task and similar instructions, the sense of agency of Mongolian students varied depending on certain other agency cues (action–effect congruency, affective valence of an effect), whereas temporal cues were only used by Austrian students (Bart et al., 2019). Thus, it seems likely that the Mongolian students understood and believed the instructions and that their sense of agency can be influenced by certain factors. However, the timing of an effect seems to be hardly relevant for Mongolian students when judging sense of agency. More specifically, neither temporal contiguity (as in this case one would expect decreasing SoA with increasing delay in both groups) nor temporal expectation (as in this case one would expect a similar data pattern as in Austrian students) are used as predominant agency cues. Nevertheless, there was a significant negative correlation between control ratings and delay in the immediate effect group and by looking at Fig. 1 it appears that the curves show a similar pattern in both cultures (even though they are considerably flatter for the Mongolian students and differences in control ratings between delays were not significant). Thus, it may not be justified to conclude that Mongolian students do not use temporal cues at all, but at least they seem to use them considerably less compared to Austrian students.

The reason why Mongolian students may pay less attention to temporal cues when inferring SoA than Austrian students may be grounded in differences in the time concept between different cultures (Block et al., 1996; Briley, 2009; Brislin & Kim, 2003; Ezzell, 2002; Fulmer et al., 2014; Levine, 1997/2006).^4^ Many Western cultures have linear time concepts, in which time moves in one direction from the past to the present to the future and past events are irretrievable (e.g., Brodowsky et al., 2008; Dahl, 1995). Accordingly, it seems reasonable that people pay attention to the timing of events, i.e., in the present context the delay after which an effect follows the action. Thus, the timing of events may be an important indicator to assess causality between events and thus SoA over events in Western cultures like Austria. In contrast, some Eastern cultures rely on cyclical time concepts with an emphasis on the reoccurrence of events (Brodowsky et al., 2008; Dahl, 1995). Accordingly, timing of events may be not attended much and thus may be also less important when inferring causality and thus SoA in Eastern cultures like Mongolia (Widlock, 2014).

Interestingly, the present results are in contrast with previous studies investigating other Eastern cultures. For instance, it has been observed that temporal contiguity is used as an agency cue in Japanese participants (cf. Kawabe, 2013; Sato & Yasuda, 2005; Wen et al., 2015). These diverging results may be explained either by methodological or by cultural differences. From a methodological viewpoint, in the studies using Japanese participants not only the action–effect delay was varied, but also other characteristics of the effect, such as congruence with a prior action (Kawabe, 2013; Sato & Yasuda, 2005) or arousal of the effect (Wen et al., 2015). It may be that in contexts with overall higher uncertainty about whether an effect is caused by oneself or not, other agency cues, such as temporal cues are more strongly relied on. However, this seems unlikely considering that also in the present experiment uncertainty was induced (by telling participants that the effect could be produced by the computer) and that in the above-mentioned studies the effect of temporal contiguity was rather consistent and did not vary depending on the effects’ congruency with prior actions (Kawabe, 2013; Sato & Yasuda, 2005) or arousal (Wen et al., 2015). Another methodological explanation may be that in the present experiment SoA was operationalized as a measure of control (i.e., indicating how much control one experienced over the effect), whereas in the above-mentioned studies it was operationalized as measure of causality (i.e., whether one caused or produced the effect, Ssato & Yasuda, 2005; Wen et al., 2015). Such wording effects may result in differences in response behavior (e.g., high temporal contiguity may be more important when judging causality compared to judgments regarding one’s own control).

From a cultural perspective, one may argue that even though many Eastern cultures share some cultural values and norms, each culture may still have its distinctive features, which may extend to differences in experiences of SoA between different Eastern cultures (the same may apply to Western cultures). For instance, in contrast to the Mongolian culture, punctuality is highly valued in the Japanese culture (at least if there are not any social obligations that are deemed as more important, Hashimoto, 2008; Steger, 2006). Thus, one may speculate that Japanese put a high emphasis on time and on timing of events and thus temporal contiguity may be an important cue for SoA. Further, one may assume that increased bilateral relations between Europe and Japan (such as trade and economic relations) have resulted in a reciprocal approximation of their cultural values, which may ultimately result in more similarities between Japanese and Western countries on different behavioral levels compared to Mongolians and Western countries.

One may raise the question whether there are circumstances under which Mongolians rely more strongly on temporal cues when assessing SoA over effects. It has been proposed that Western and Eastern cultures think in different units of time. For instance, regarding punctuality, it has been observed that Western cultures often think in units of five minutes, whereas in many Eastern cultures the unit of time is 15 min. Accordingly, people who arrive 10 min after the agreed meeting time would be perceived as being late in Western cultures, whereas this is not so much the case in many Eastern cultures (Brislin & Kim, 2003). One may speculate that in a similar way cultures think in different units of time when assessing temporal contiguity. For instance, it may be possible that in contrast to Austrian students, Mongolian students may still perceive actions and effects that are separated by a 600 ms delay as close together in time. Accordingly, in the present paradigm they may not have perceived much uncertainty about whether they caused the effect, thus experienced a high SoA over all effects independently from the action–effect delay (as can be also seen in Fig. 1) and therefore hardly used temporal agency cues to infer SoA over effects. One may speculate that with larger action–effect delays (e.g., in the range of seconds), Mongolians would experience a higher uncertainty about whether they caused the effect and thus would rely more strongly on temporal agency cues.

In any way, the present results clearly indicate that Austrian students and Mongolian students differ in their use of temporal cues to assess SoA over effects. This is in line with previous studies which already indicated that not only similarities, but also differences between Western and Eastern cultures exist in SoA (Aarts et al., 2010; Barlas & Obhi, 2014; Bart et al., 2019) and that not all agency cues may be used universally (Bart et al., 2019). Further, this may fit well with cue integration models of SoA (Farrer et al., 2013; Synofzik et al., 2008, 2009, 2013) according to which different cues are weighted and integrated according to their reliability in a given situation in order to determine whether oneself or someone else is responsible for a certain action/effect (Moore & Fletcher, 2012; Synofzik et al., 2009). In Mongolian students SoA ratings were quite high in both, the immediate and the delayed group suggesting that the SoA of Mongolian students is hardly impaired by deviations in the timing of an effect (corresponding to a previous study, Bart et al., 2019). Presumably, they perceive temporal cues as less reliable (e.g., due to time concepts according to which timing of events is less important) and therefore may not use them or at least use them to a lesser extent to infer SoA over effects compared to Austrian students. One may raise the question which cues Mongolians use instead. In a previous study it has been observed that, similar to Austrian students, Mongolian students seem to use action–effect congruency and affective valence of an effect (i.e., more SoA for positive compared to negative effects) as agency cues (Bart et al., 2019). Further, there may be various other agency cues (e.g., one’s prior intentions or beliefs, or proprioceptive cues, cf. Synofzik et al., 2008, 2009), which so far have not been investigated in Mongolians. A tentative speculation is that cultural values affect the perceived reliability of different agency cues and that thus cue integration is culture-depended.

A limitation of the present study is that we investigated student samples, which are not representative for the whole population of a country. In particular, Mongolian students may be a special group as they live in Ulaanbatur, the capital and largest city of Mongolia, which becomes more and more industrialized and urbanized. Thus, their lifestyles and correspondingly their values, norms and most importantly their concepts of time may differ from people living in more rural areas. Accordingly, it may be of interest for future studies to investigate whether time concepts and thus temporal cues to infer SoA over effects differ between urban vs. rural subsamples in Western and Eastern cultures. A further limitation is that we investigated only Mongolian students as representatives for Eastern cultures. Results obtained in Austrian and Mongolian students may not be directly generalized to other Western or Eastern cultures. For instance, as mentioned above, temporal contiguity seems to be used as agency cue in Japanese participants (cf. Kawabe, 2013; Sato & Yasuda, 2005; Wen et al., 2015). Thus, future studies may compare a wider variety of different Western and Eastern cultures. Additionally, future studies may investigate cultural differences in time perception processes (e.g., temporal binding; Haggard et al., 2002; or temporal recalibration, Stetson et al., 2006) and relate them to the use of temporal cues to get a more thorough understanding of why temporal cues may be less likely used to infer SoA over effects in certain cultures.

In conclusion, temporal expectation and not temporal contiguity is used as predominant agency cue in Western cultures, whereas Eastern cultures hardly rely on temporal cues when inferring SoA over effects. Thus, taken together, even though SoA may be a universal concept, the use of certain agency cues depends on culture.

## Data Availability

The necessary data and materials to reproduce the reported results are available at the Open Science Framework (OSF), https://osf.io/kuemv/.

## Footnotes

1 Participants in the immediate effect group performed two further adaption blocks at the end. This was done simply for logistic reasons. Because of the short action-effect delay in adaption blocks and in more than half of the trials in the test blocks in this group, participants would have finished the experiment faster than the delayed effect group. However, we wanted to ensure that participants of both groups finish the experiment at roughly the same time due to the group testing situation.

2 Note that we also performed the analyses including all 30 trials in the 0 ms and 600 ms condition, respectively. Results were essentially the same.

3 We chose longer delays because we used visual instead of auditory effects. As the temporal processing and perception of interval duration is less accurate with visual compared to auditive stimuli (e.g., Burr et al., 2009; Repp & Penel, 2002), we had to ensure that our delays were long enough such that participants would still perceive them as temporally distinct from each other.

4 As an anecdotal footnote: The author ES gave an example showing that the conception of time is very different in Mongolian students compared to Austrian students. Students in Mongolia are often late for their exams, which is why exams do not start on time but as soon as half of the students are present.
